# Chitosan-Doped TiO_2_ Functionalized Asphalt Mixtures for NO_2_ Mitigation Under High Pollution Levels

**DOI:** 10.3390/ma18184292

**Published:** 2025-09-12

**Authors:** Amanda Pontes Maia Pires Alcantara, Larissa Virgínia Silva Ribas, Débora Barbosa Silva, Jairo Ivo Castro Brito, Elisabete Fraga Freitas, Francisco Wagner Sousa, Verônica Teixeira Franco Castelo Branco

**Affiliations:** 1 Department of Transportation Engineering, Federal University of Ceará, Fortaleza 60440-900, Brazil; amanda.a@det.ufc.br (A.P.M.P.A.); larissa.ribas@det.ufc.br (L.V.S.R.); jairoivo.brito@det.ufc.br (J.I.C.B.); 2 Department of Transportation and Geodesy Engineering, Federal University of Bahia, Salvador 40210-630, Brazil; 3 Advanced Production and Intelligent Systems Associated Laboratory (ARISE), Department of Civil Engineering, Institute for Sustainability and Innovation in Structural Engineering (ISISE), University of Minho, Campus Azurém, 4800-058 Guimarães, Portugal; 4 Department of Chemistry, Federal Institute of Ceará, Caucaia 60040-215, Brazil; fr.wagner@ifce.edu.br

**Keywords:** photocatalytic pavements, chitosan–TiO_2_, alternative photocatalytic reactor, biomaterials, adhesion

## Abstract

Improving air quality is a significant environmental challenge. This research explored the potential of asphalt mixtures functionalized with a chitosan–TiO_2_ composite (CS-TiO_2_) to reduce high NO_2_ concentrations and improve durability. For the assessment of the photocatalytic efficiency of the new CS-TiO_2_ composite, a low-cost reactor adapted to accommodate asphalt Marshall-type specimens and high pollutant concentrations encompassing a passive sampling module was developed. The CS-TiO_2_ was synthesized using a wet impregnation method at a concentration of 2%, and asphalt mixtures were treated with aqueous solutions of the photocatalysts at 2.5 g/m^2^ and 5.0 g/m^2^. Laboratory tests using the photocatalytic reactor and passive sampling of NO_2_ revealed pollutant reductions of 21% with TiO_2_ and 28% with CS-TiO_2_. CS-TiO_2_ achieved 15% efficiency in visible light, reducing NO_2_ levels and offering UV protection to the asphalt mixtures. Additionally, the chitosan improved the photocatalyst’s adhesion by about 18%, as confirmed by tape test results, suggesting enhanced durability on pavement surfaces. The results achieved showcase the relevance of the proposed methodological improvements for supporting further research.

## 1. Introduction

The growing concern over air quality in urban areas has driven the search for solutions to mitigate atmospheric pollutants. Among these pollutants, nitrogen dioxide (NO_2_) stands out due to its adverse effects on human health and the environment [1]. NO_2_ is a critical air quality standard as it directly influences the formation of photochemical smog and particulate matter (PM_2.5_), as well as being a precursor to tropospheric ozone (O_3_) [1,2]. Moreover, there is a strong relationship between transport systems and air pollution. The transportation sector generates over 25% of global CO_2_ and 78% of NO_x_ emissions [1]. High concentration and prolonged exposure to NO_2_ are associated with respiratory and cardiovascular diseases, making its mitigation a priority in environmental policies [3,4].

Environmental catalysis can become a crucial tool for promoting improvements in air quality. In this context, advanced oxidation processes (AOPs) are traditional in environmental depollution studies of aqueous matrices and have also been employed in research for the purification of industrial and agricultural gases [5,6,7]. AOPs are promising due to their reliability, cost-effectiveness, and safety as air depollution methodologies, as they directly degrade pollutants into less harmful substances without requiring additional steps [8].

Photocatalysis is a specific type of AOP in which a semiconductor material (or photocatalyst) converts solar energy into chemical energy to oxidize and reduce substances, transforming them into more useful forms. Among the existing semiconductors, TiO_2_ is one of the most used due to its strong oxidation capacity, ability to decompose organic pollutants, chemical stability, durability, non-toxicity, low cost, and availability in nature [9,10,11]. However, its energy absorption occurs preferably within the UV range (*Eg* = 3.2 eV ≈ 390 nm), which accounts for less than 5% of the solar spectrum. This limitation reduces the efficiency of these materials in applications utilizing natural light [12,13,14]. To overcome these limitations, surface modifications are often made to these materials through semiconductor doping to produce visible-light-sensitive photocatalysts [15].

Asphalt pavements are a suitable location for mitigating atmospheric pollutants due to their proximity to vehicle traffic and the extensive urban areas available [16,17,18]. This can be achieved by functionalizing these structures with semiconductor materials that act as photocatalysts to convert pollutants into less harmful substances, thereby reducing their availability in the atmosphere [19].

The semiconductor material can be incorporated into the asphalt mixture at two main stages of its construction: during asphalt mixture production or after compaction [20,21,22]. During production, photocatalysts can be included in the asphalt mixture as aggregates (filler) or dispersed in the asphalt binder. After compaction, they can be sprayed or spread as solutions on the pavement surface.

Functionalization after compaction typically results in a thin film deposited on the road’s surface. This approach improves radiation absorption and the adsorption of pollutant substances by the semiconductor, allowing for their chemical modification. This method achieves higher pollutant mitigation percentages than incorporation into the asphalt mixture; however, it also incurs significant losses of semiconductor material due to the low adhesion of photocatalysts to the pavement. In these types of functionalization, adhesion is primarily achieved through the physical anchoring of particles due to the natural roughness of pavement surfaces, with losses mainly occurring due to the modification of this surface layer caused by wind, rain, and traffic [17,23].

Besides TiO_2_, other types of photocatalysts have been tested to mitigate atmospheric pollutants using pavements as a substrate, including ZnO [24], g-C_3_N_4_ [25,26,27], TiO_2_/CeO_2_ composites [28], TiO_2_-Al_2_O_3_ [29], g-C_3_N_4_/TiO_2_ [25], as well as TiO_2_ doped with graphene [30] and metals like iron [31,32], copper [32,33], cerium [32,34], silver [35], and lanthanum [36]. Nevertheless, optimizing TiO_2_ doping strategies to enhance its photocatalytic performance remains an open field of research, in which the use of biobased materials presents a promising alternative due to their environmental appeal.

Environmental awareness has driven the search for green, renewable materials with less environmentally harmful processing routes. TiO_2_ can be incorporated into different organic matrices to form hybrid compounds with industrial and environmental potential [37]. Chitosan (CS) is an abundant biopolymer that has attracted attention in various fields due to its biodegradability, low cost, and wide applicability in composite materials [27,38].

Among the characteristics of CS, its adhesive properties are noteworthy, favoring its application as a bioadhesive [39,40] and its chemical compatibility with TiO_2_, which is used in composites for the photodegradation of aqueous pollutants (organic dyes) [41]. It has also been used to modify asphalt binder to produce asphalt emulsions [42,43]. These characteristics support the hypothesis that chitosan–TiO_2_ (CS-TiO_2_) composites could add new functionalities to asphalt mixtures, in terms of catalytic performance and adhesion improvement. Another relevant challenge concerns the methods used to evaluate or quantify photocatalytic efficiency.

The photocatalytic activity of these structures is commonly assessed in the lab through direct and indirect methods. The indirect method evaluates the efficiency of photocatalytic pavements immersed in aqueous solutions of organic dyes such as Rhodamine B, using photoreactors [23,34,44,45]. Despite its historical relevance, this approach is limited by the fact that it does not operate in the gas phase or involve the target pollutants (or adsorbates). The physicochemical properties of the species involved in the photocatalytic process influence the adsorbate–substrate interaction and, consequently, the surface dynamics during chemical reactions. This can lead to biased interpretations of results and misrepresent the expected performance of the technology in more realistic contexts.

The analysis of reaction products, or of the remaining concentration of the input pollutant, using chemiluminescence devices, chromatography, or electrochemical analyzers connected to the outlet of the photoreactors, is also a common approach in the testing of photocatalytic materials [35,46,47,48,49,50]. However, these analyses are time-consuming (in the case of chromatographic methods), costly, require specialized technical support, and cannot be conducted in situ where the materials are applied or during the actual service life of the structure. These analytical techniques are expensive and unsuitable for analyzing or quantifying pollutants—and therefore the photocatalytic process—in real-world scenarios.

Scientific efforts to develop alternative methods to certified protocols [49,50,51] for testing photocatalytic surfaces and evaluating their efficiency are diverse. For example, photocatalyst activity indicator inks (PAIIs) were developed using redox indicator dyes applied to photocatalytic surfaces. The change in color or discoloration correlates with the surface’s photoactivity and can be used in situ without requiring specialized equipment [52,53]. However, PAIIs are considered more qualitative than quantitative.

Other experimental setups have also been developed to facilitate photocatalytic testing in laboratories and to better align with specific research objectives [18,54,55,56]. The photocatalytic reactors currently available in the literature—including the ISO standard [50]—present significant limitations in terms of cost and adaptability to alternative analytical methods. Therefore, there is a clear need to develop an alternative photocatalytic reactor that prioritizes cost reduction and compatibility with diverse analytical techniques; it should be optimized for asphalt samples, such as Marshall specimens, and capable of treating various pollutant gases (NO_x_, SO_2_, and vehicle exhaust) at different concentrations. Therefore, there is a clear need to develop an alternative photocatalytic reactor that prioritizes cost reduction and compatibility with diverse analytical techniques.

In the laboratory, the concentration of the semiconductor and the influence of meteorological parameters can be simulated and evaluated using photocatalytic reactors specifically designed for this purpose. Several studies in the literature utilize ASTM D5116 [49], ISO 22197-1 [50] and JIS R 1701-1 [51], and photoreactors or adaptations thereof to test the performance of materials developed or synthesized for atmospheric pollutant mitigation [30,35,46,47,48].

Adapted photoreactors aim primarily to reduce the experimental costs of research, adapt the experimental conditions for the tested materials based on the understanding of the phenomenon, and vary relevant conditions for each use, creating alternative routes to obtain insights into the properties of tested materials during the laboratory phase.

The review performed unveils several main research gaps, such as the limited adhesion of photocatalytic films to asphalt surfaces, the low visible-light absorption of TiO_2_, the scarcity of biobased dopants that enhance both photocatalytic and adhesive properties, and the absence of cost-effective and versatile testing setups for photocatalytic efficiency under realistic gas-phase conditions. Moreover, current evaluation methods often do not reflect actual field conditions, making it difficult to assess the true performance of photocatalytic materials applied to pavements. This research aims to enhance the state of the art on: the development of hybrid chitosan–TiO_2_ composites to improve photocatalytic degradation of NO_2_ under simulated high-pollution environments; the adhesion performance of these composites when applied to asphalt pavements; the design and application of a low-cost, adaptable photocatalytic reactor compatible with different pollutant gases and analytical methods; and provide answers to the following questions:

- Can chitosan doping improve both surface adhesion and the photocatalytic activity of TiO_2_ when applied to asphalt pavements?
- How effective is the proposed functionalization in reducing NO_2_ concentrations under controlled gas-phase conditions using the adapted reactor?

The expected impacts are environmental and societal by using eco-friendly solutions and reducing harmful pollutants in the air through an accessible and scalable photocatalytic strategy. The proposed chitosan–TiO_2_ composite not only contributes to urban air quality improvement by targeting NO_2_, a critical and hazardous pollutant, but also incorporates a renewable biopolymer, aligning with circular economy and green chemistry principles.

Additionally, by developing a low-cost and customizable reactor for pollutant abatement tests, this research democratizes access to advanced testing methodologies, especially for research groups with limited resources. The outcomes may support public policy and infrastructure planning focused on sustainable urban mobility and climate change mitigation, especially in densely populated or high-traffic regions facing air quality challenges.

## 2. Materials and Methods

### 2.1. Overview

**Figure 1:**
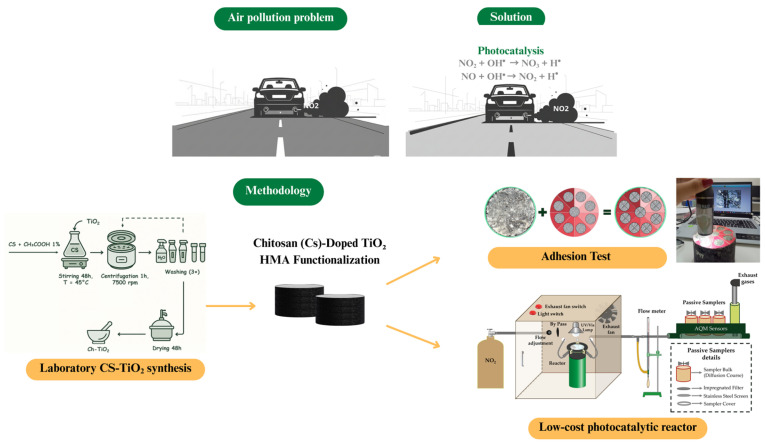
Method Summary Flowchart.

The following methodology was developed to investigate the research questions mentioned in the previous section (introduction). A schematic flowchart illustrating the overall methodology is provided in Figure 1 to facilitate the visualization of the sequential steps. Initially, the TiO_2_–chitosan composite was synthesized and characterized. These specimens were then functionalized by applying a solution containing the synthesized composite. Subsequently, the specimens were evaluated for adhesiveness, using the adhesion test, and for their photocatalytic activity in degrading high concentrations of NO_2_, employing an alternative, low-cost reactor specifically designed for this purpose.

### 2.2. Synthesis and Characterization of the CS-TiO_2_ Composite

A commercial high degree of purity TiO_2_ (Eleven Box^®^, Barueri, SP, Brazil) in the anatase phase was modified with low-molecular-weight chitosan (CS; 50,000–190,000 Da or >75%) (Sigma-Aldrich^®^, St. Louis, MO, USA) to produce the CS-TiO_2_ composite. The CS was incorporated into the TiO_2_ at a concentration of 2%, following an adapted methodology from Zubieta et al. [57], who utilized a wet impregnation method for surface modification of TiO_2_ with CS.

**Figure 2:**
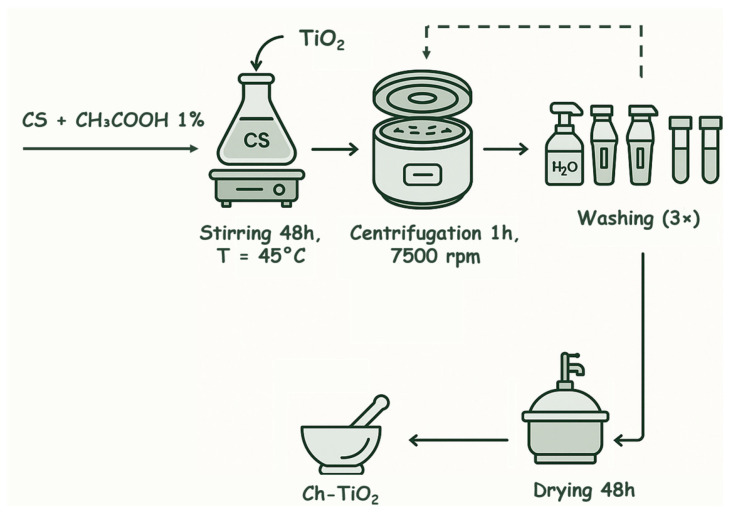
Flowchart of CS-TiO_2_ synthesis steps.

Therefore, CS was dispersed in 20 mL of diluted acetic acid (CH_3_COOH, 1% *v*/*v*; Dinamica^®^, Indaiatuba, SP, Brazil) to achieve a 2% (*w*/*w*) CS concentration in TiO_2_. After the complete dissolution of CS, 1 g of TiO_2_ was added, and the mixture was stirred vigorously at 45 °C for 48 h. The mixture was then centrifuged at 7500 rpm for 1 h (Centrifuge 5430; Eppendorf^®^, São Paulo, SP, Brazil) to achieve phase separation and isolate the synthesized composite. After centrifugation, the supernatant was removed, and distilled water (H_2_O) was used for sequential washing of the material to eliminate residual acid. The centrifugation process was repeated after each wash. The final solid (CS-TiO_2_) was dried in a vacuum desiccator for 48 h and subsequently ground to achieve disaggregation. The final product was not calcined to preserve the CS within the material’s composition. Figure 2 presents the process flow for the synthesis of the CS-TiO_2_ composite.

A set of analytical techniques was employed to characterize the synthesized composite. The surface morphology of the samples was analyzed using scanning electron microscopy with an energy dispersive detector (SEM-EDX) (Quanta 450 FEG, FEI^®^, Hillsboro, OR, USA). Structural analysis was conducted using X-ray diffraction (XRD) in powder mode with a D8 Advance diffractometer (Bruker Optics^®^, Billerica, MA, USA), using CuKα radiation (λ = 1.5406 Å), at 40 mA and 40 kV in Bragg–Brentano geometry. For particle size distribution and zeta potential measurements, 0.25 mg of TiO_2_ or CS–TiO_2_ was dispersed in 10 mL of ultrapure water (0.025 mg/mL), followed by 10 min of sonication. Particle size was measured using 900 μL of each suspension in polystyrene cuvettes (1 cm × 1 cm), while zeta potential was determined using capillary cuvettes (DTS1070, Malvern Panalytical^®^, Malvern, Worcestershire, UK), both analyzed in a Zetasizer Nano ZS (Malvern Panalytical^®^, Malvern, Worcestershire, UK). FT-Raman spectroscopy was performed using compressed powder samples in a VERTEX 70 spectrometer (Bruker Optics^®^) equipped with a Ram II module and an infrared laser (1064 nm). Scattered light was detected by a germanium detector, and spectra were recorded from 40 to 4000 cm^−1^ at room temperature to capture both low- and high-wavenumber Raman-active modes. Finally, FTIR spectroscopy was carried out in vacuum using a VERTEX 70 V spectrometer (Bruker Optics^®^, Billerica, MA, USA) with a Globar source for mid-infrared (MIR) and DLaTGS detectors. The system featured a HeNe laser (λ = 633 nm) for optical path calibration, operated at 2 cm^−1^ resolution with 128 scans and a wide-range Si beam splitter for MIR analysis. The use of vacuum enhanced the detector sensitivity.

### 2.3. Functionalization of Asphalt Mixture Specimens

The specimens used for functionalization consisted of hot mix asphalt (HMA) with a binder content (penetration grade 50/70 bitumen) of 4.5%. Aqueous solutions for functionalizing the specimens were prepared to deposit either 2.5 g/m^2^ or 5.0 g/m^2^ of the photocatalyst onto the surface. These application rates were selected based on previous literature and were inspired particularly by Wang et al. [54], who evaluated these concentrations as optimal for the degradation of vehicle exhaust gases using TiO_2_. Therefore, 0.08 g or 0.16 g of TiO_2_ or CS-TiO_2_ were dispersed in 2.5 mL of distilled water, and the solutions were applied using a paintbrush for surface coating.

### 2.4. Adhesion Test Method

To test the adhesion of the photocatalysts to the specimens, an adapted “tape test” method was used, based on ASTM D3359 [58] and ISO 2409 [59] standards. These standards assess the area detached from a coating film after abrupt peeling using adhesive tape applied over an “X” incision marking the evaluation area. There are no sufficiently rigorous tests for this specific purpose; however, the proposed method, although quick, is used in other areas and has already been used on pavements as well.

**Figure 3:**
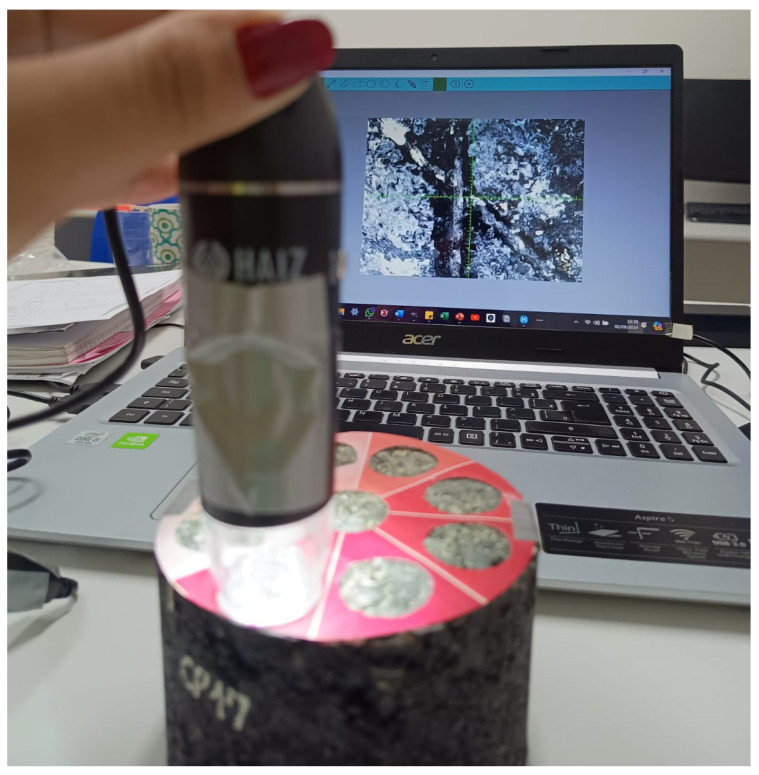
Digital microscope positioning and image capture for DIP.

The test method involved evaluating the coating’s adhesion to the substrate by peeling off a transparent adhesive tape (model: polyester filament tape; brand: tesa SE^®^, Norderstedt, Schleswig-Holstein, Germany) with a width of 25 mm and adhesion strength of 9 N/cm on a steel plate. The specimen surfaces were analyzed using digital image processing (DIP). Images were obtained before and after the tape test using a USB digital microscope (model: Hz-1600X; brand: HAIZ^®^, Indaiatuba, SP, Brazil) (Figure 3). All images were captured using the device’s software (HiView20230724^®^, HAIZ^®^, Indaiatuba, SP, Brazil).

**Figure 4:**
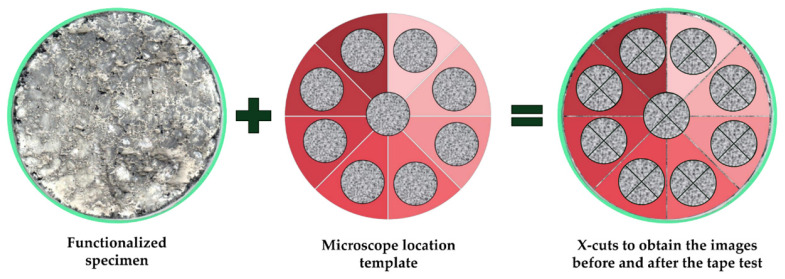
Flowchart for preparing the samples for the tape test.

To standardize the area analyzed in DIP for the specimens, a cylindrical template with nine internal circles of 22 mm in diameter was used to capture images at the center of the “X” incision (Figure 4). The standards recommend choosing an area with minimal roughness for making the “X” incision, where the filament tape is applied for the peeling test and visual analysis of the detached area. Therefore, the internal circles were designed to select the ideal area for performing the “X” incision and adhesion test.

Subsequently, ImageJ^®^ software (version 1.54g, National Institutes of Health, Bethesda, MD, USA). was used to calculate the percentages of the area initially covered by the photocatalyst and the remaining content on the specimen after the tape test, quantifying the area removed. DIP analyses have already been used in studies on the adhesion of materials, both for paints [60] and binder–aggregate systems [61].

Three images were taken in each chosen circle, and the microscope was repositioned between each capture. This procedure was repeated for three circles for each specimen. Additionally, the zoom, focus, and lighting of the microscope were kept constant during all image acquisitions. The photocatalyst loss was measured based on an adaptation of the adhesion test classification table outlined in ASTM D3359 [58].

### 2.5. Photocatalytic Testing Setup

The photocatalytic efficiency of the tested materials in reducing NO_2_ was assessed using a photocatalytic reactor specifically designed to analyze functionalized asphalt pavement samples. The reactor was adapted from ISO 22197-1 [50] to test cylindrical HMA specimens (Marshall-type) and to modify gas analysis and quantification methods, since acquiring a standardized reactor is generally expensive. Additionally, ISO-type reactors generally use rectangular reaction chambers; therefore, they are unsuitable for testing ordinary cylindrical specimens, which are most used in dosage, mechanical characterization, and extraction of field samples of HMA [62,63].

**Figure 5:**
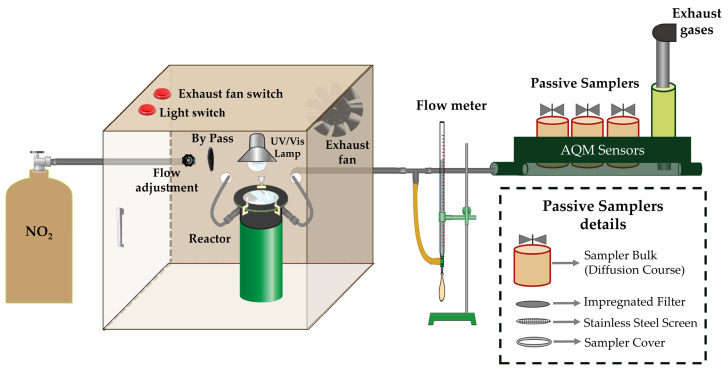
Photocatalytic reactor layout.

The system (Figure 5) included a NO_2_ cylinder with a standard concentration of 500 ppm NO_2_ balanced in helium (NO_2_/He), a nitrogen (N_2_) cylinder for diluting the input gas, a bubbler flask for humidity simulation, a cylindrical stainless-steel reaction chamber with a quartz window, UVC (5 W) and visible (25 W) lamps, a thin-film linear flow meter, flow control valves, and an exhaust unit. The specimen and quartz window were separated by a 5 mm ± 0.5 mm air gap through which the test gas flowed. Additionally, cost-effective methods were employed to monitor the photocatalytic reaction in the laboratory. The high-cost chemiluminescence analyzers were replaced with a multi-component detection system capable of using simultaneous qualitative and quantitative methods at medium/low costs. This system integrates several passive samplers and an air quality monitor (AQM) with low-cost sensors. The reactor was constructed using materials with low adsorption and UV resistance properties.

The passive sampling of NO_2_ was adapted from the methodologies proposed by [63,64,65,66,67]. Cellulose filters with a diameter of 25 mm were pre-washed in an ultrasonic bath using distilled water (H_2_O) and, subsequently, with methanol (MetOH) (NEON^®^, Suzano, SP, Brazil), in triplicate for both solvents. After drying, the filters were impregnated with 100 µL of a selective absorbing solution for NO_2_, composed of 0.5 mol·L^−1^ potassium iodide (KI) (Dinamica^®^, Indaiatuba, SP, Brazil) and 0.2 mol·L^−1^ potassium hydroxide (KOH) (Dinamica^®^, Indaiatuba, SP, Brazil) in methanol (NEON^®^, Suzano, SP, Brazil). Following complete drying in a vacuum desiccator, the filters were placed into the samplers housed in the reactor’s outlet box.

In a subsequent step, NO_2_ determinations on the collected filters were performed using a spectrophotometric method adapted from Saltzman [68]. Each filter was transferred to a Falcon tube, and 6 mL of a Griess–Saltzman reagent solution was added. This solution consists of 0.5% (*w*/*v*) sulfanilamide (NEON^®^, Suzano, SP, Brazil), 9% (*v*/*v*) phosphoric acid (H_3_PO_4_(Dinamica^®^, Indaiatuba, SP, Brazil), and 0.005% (*w*/*v*) N-(1-naphthyl)-ethylenediamine dihydrochloride (NEDA) (Sigma-Aldrich^®^,St. Louis, MO, USA).

The tubes containing the filter and reagent solution were homogenized using a vortex mixer (model: K45-2810 KASVI^®^, Pinhais, PR, Brazil) for 20 s. After 15 min, the supernatant was analyzed using a UV-Vis spectrophotometer (model: Specord 250 Analytik Jena^®^, Jena, Thuringia, Germany), configured to read the samples at 540 nm. The concentrations of NO_2_ in the monitored environment were calculated by applying Fick’s law according to Equation (1), where ***C*** is the concentration of NO_2_ in the air (µg·m^−3^), ***m*** is the mass of the analyte obtained from the UV-Vis analysis (µg), ***L*** is the length of the diffusion path (m), ***D*** is the molecular diffusion coefficient of NO_2_ (m^2^·h^−1^), ***A*** is the cross-sectional area of the passive sampler (m^2^), and ***t*** is the sampling time (h).

(1) $$C=\frac{m\times L}{D\times A\times t}$$

The AQM was developed at the Transportation and Environment Laboratory (TRAMA) at the Federal University of Ceará. The AQM includes calibrated sensors to measure NO_2_, temperature, and humidity: the MiCS-6814 sensor (SGX Sensortech^®^, Shanghai, China) for NO_2_ detection and the BME280 sensor (Bosch^®^, Gerlingen, Baden-Württemberg, Germany) for temperature and humidity measurements (Table 1).

For the photocatalytic tests, fixed parameters of NO_2_ concentration (500 ppm), humidity (50%), temperature (25 °C), irradiance (20 W/m^2^), and gas flow rate (100 mL.min^−2^) were set. The initial NO_2_ concentration was set at 500 ppm to test efficiency under extreme gas concentration conditions, well above current legislation; humidity (%) was set at 50% because it is the typical minimum humidity for the city of Fortaleza during the dry season. The temperature was set at 25 °C because increasing the temperature could alter the chemical composition of the asphalt binder, and irradiance was set at 20 W/m^2^ to ensure adequate photocatalyst activation and avoid excessive thermal effects. The reactor gas flow rate was set at 0.1 L.min^−1^ based on the findings of the study by Mikyskova et al. [69], which demonstrated higher photocatalytic efficiency at lower space velocities than that proposed by ISO 22197-1 [50], which is 3 L.min^−1^. Additionally, two photocatalytic materials—TiO_2_ and CS-TiO_2_—were tested at two different application rates (2.5 and 5.0 g.m^−2^) under two radiation wavelengths (UV and visible) to assess the influence of flow and radiation on pollutant reduction. The influence of these variables on the pollutant degradation efficiency of the photocatalytic pavements was evaluated using a factorial design, optimizing experimental conditions to assess the effects of variables on NO_2_ conversion simultaneously (Table 2).

The photocatalytic conversion was subsequently calculated according to Equation (2), where: ***η*** is the NO_2_ conversion efficiency; ***C_i_*** and ***C_f_*** represent the concentration of the pollutant (in ppm) at the beginning (after 1 h without exposure to light) and at the end (after 1 h of exposure to light) of the photocatalytic process, respectively.

(2) $$\eta \left(\%\right)=\frac{\left(C_{i}-C_{f}\right)}{C_{i}}\times 100$$

## 3. Results

### 3.1. Characterization of the Synthesized Photocatalysts

**Figure 6:**
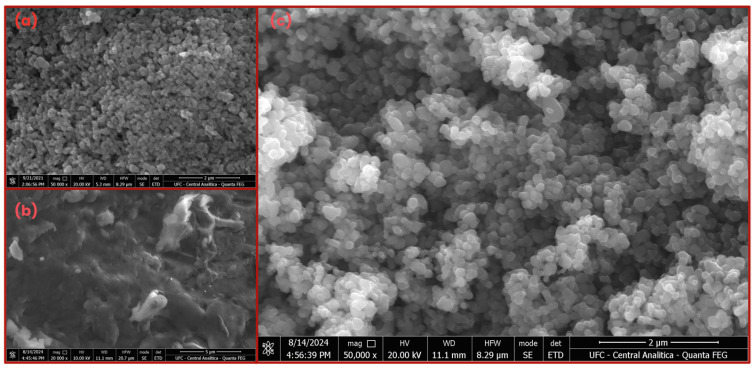
SEM image of (**a**) TiO_2_, (**b**) CS, and (**c**) CS-TiO_2_.

Figure 6 presents SEM images of TiO_2_, CS, and CS-TiO_2_, respectively, showing the shape and size of the materials. The morphology of TiO_2_ and CS-TiO_2_ particles is spherical-like, while CS appears to have a rougher and more amorphous surface. The average particle size of TiO_2_ and CS-TiO_2_ was approximately 80 and 190 nm; however, CS-TiO_2_ exhibited a few larger particles, ranging from 300 to 400 nm. Despite this, the analysis indicates that the synthesis slightly alters the nanometric profile of the CS-TiO_2_ composite. Additionally, the spherical profile of the composite suggests that CS may have attached to the surface of TiO_2_ in a core–shell-like structure, like the findings reported by [57,70,71].

**Figure 7:**
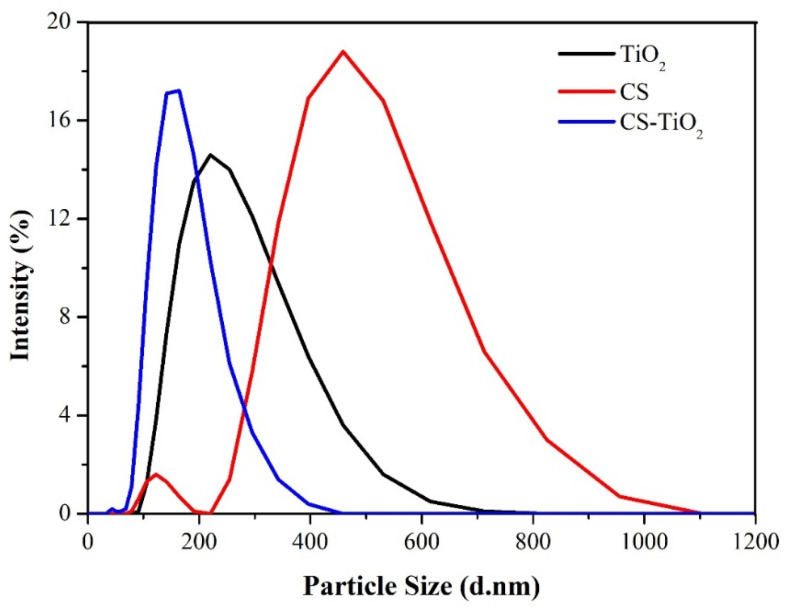
Particle Size Distribution of the Photocatalysts.

Figure 7 shows the results of DLS analysis and the particle size distribution of TiO_2_, CS, and CS-TiO_2_. Unlike SEM analysis, DLS measures particle size when dispersed or dissolved in a liquid, in this case, H_2_O. The average particle sizes measured for TiO_2_, CS, and CS-TiO_2_ were 217.6 nm ± 114.4 nm, 579.9 nm ± 136.8 nm, and 183.6 ± 559 nm, respectively.

**Figure 8:**
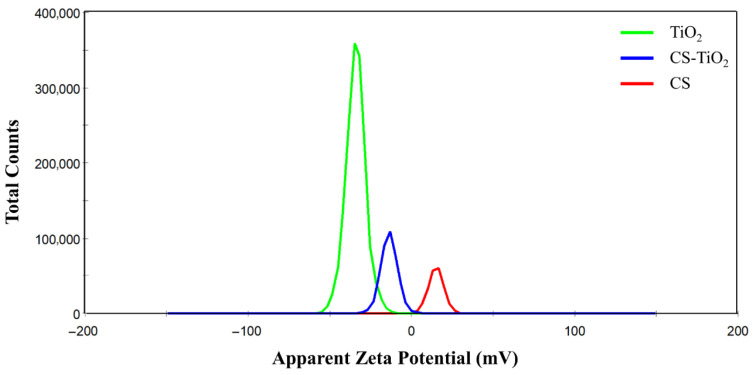
Zeta Potential of TiO_2_, CS, and CS-TiO_2_.

Figure 8 presents the zeta potential analysis results. The samples displayed distinct surface charges in ultrapure H_2_O. TiO_2_ exhibited a negative zeta potential with an average value of −341 ± 62 mV. CS showed a positive zeta potential with an average value of 138 ± 46 mV, while CS-TiO_2_ had a negative zeta potential of −136 ± 52 mV.

The magnitude of the zeta potential indicates the stability of the colloidal system. If the suspended particles have a high positive or negative zeta potential, they tend to repel each other, reducing the tendency to flocculate. Conversely, if the zeta potential is low, there are insufficient repulsive forces to prevent particles from aggregating and flocculating. The general threshold between stable and unstable suspensions is conventionally set at ±30 mV. Therefore, the obtained values show that TiO_2_ and CS-TiO_2_ tended to flocculate in aqueous formulations.

Moreover, the zeta potential values indicate that, when TiO_2_ is combined with CS, an interaction between the opposite charges of CS (positive) and TiO_2_ (negative) may occur, potentially forming ionic bridges, as suggested by Wiącek et al. [72]. This process is consistent with the increase in the magnitude of the zeta potential to −136 mV in the CS-TiO_2_ composite compared to TiO_2_ alone. This change suggests partial coverage of TiO_2_ particles by CS chains in a system stabilized by electrostatic and steric forces. This electrosteric stabilization was discussed by Hussein et al. [73], who reported similar effects of CS interaction with TiO_2_ particles.

**Figure 9:**
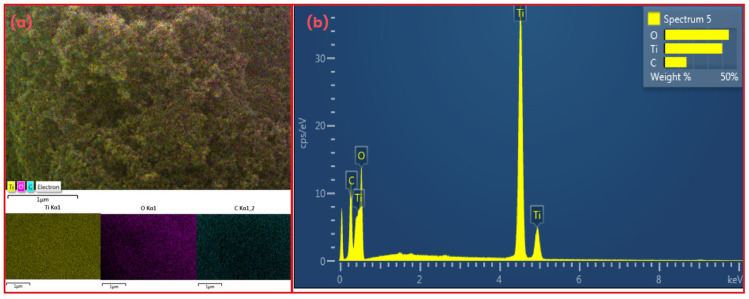
Images (**a**) and EDX spectrum (**b**) of TiO_2_.

Figure 9 presents the elemental analysis of the TiO_2_ sample obtained by EDX. The presence of carbon is attributed to the support material used during sample preparation. The images and spectra confirm that the TiO_2_ sample is composed only of titanium (Ti) and oxygen (O). These results indicate the high purity of the TiO_2_ employed in the synthesis of the CS-TiO_2_ composite.

**Figure 10:**
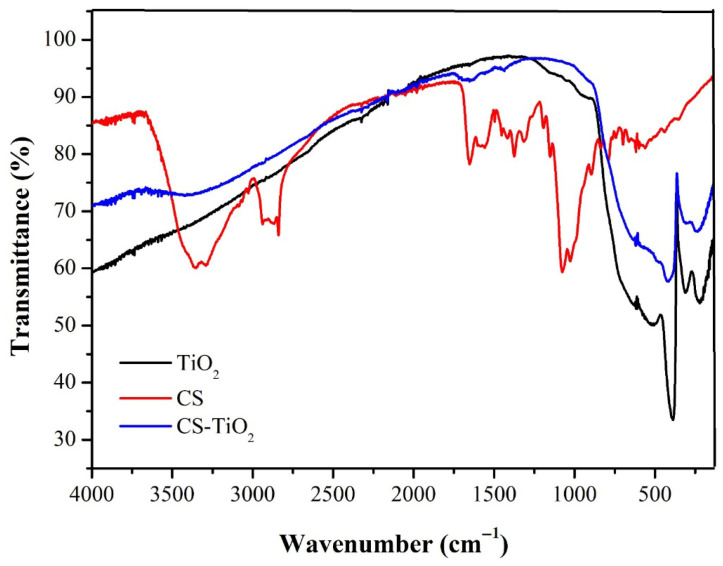
FTIR Spectra of TiO_2_, CS, and CS-TiO_2_.

Figure 10 shows the FTIR spectra of TiO_2_, CS, and CS-TiO_2_ samples. The bands around 800 and 200 cm^−1^ correspond to the O-Ti-O stretching vibrations of TiO_2_; note that the intensity of these bands is reduced in the CS-TiO_2_ spectrum. The bands around 3360 and 3000 cm^−1^ correspond to the O-H and N-H stretching vibrations present in CS, respectively. The bands around 1650 and 1540 cm^−1^ correspond to the C=O stretching and N-H in-plane bending vibrations, respectively. They are associated with CS amides. Finally, the bands around 1060 and 1000 cm^−1^ correspond to C-O stretching in CS. These key vibrations from the CS and TiO_2_ spectra are also present, albeit subtly, in the CS-TiO_2_ spectrum, confirming the presence of CS and TiO_2_ in the synthesized photocatalyst. Spoială et al. [70] obtained similar FTIR spectra and results in their studies on CS-TiO_2_ synthesis.

The combined analysis of the results corroborates the formation of the CS-TiO_2_ composite via the proposed synthesis route. The composite, therefore, integrates the chemical and structural properties of both TiO_2_ and CS consistently.

### 3.2. Evaluation of Photocatalyst Film Adhesion to the Specimen Surface

The images captured before and after the tape test were processed using ImageJ^®^. The pre-test image was subtracted from the post-test image to isolate the detached photocatalyst layer. A thresholding technique was then applied to enhance contrast based on pixel intensity, allowing for the identification of the peeled areas while minimizing background noise. The number of pixels corresponding to the detached region was quantified to estimate the percentage of material removed, indicating the adhesion performance of the photocatalyst. The DIP results were analyzed for outliers (using boxplots) and measures of central tendency and dispersion. Table 3 shows the results for each photocatalyst evaluated in this study.

Comparing the types of photocatalyst solution between them, it can be observed that the percentage of material detached from the specimen’s surface is practically the same, regardless of the concentration. However, the CS-TiO_2_ formulations show a slight improvement of 18.1% and 10.5% in adhesion for CS-TiO_2_ at 2.5 g/m^2^ and 5.0 g/m^2^, respectively, compared to the TiO_2_ films.

Thus, from an adhesion standpoint, the CS-based formulations were considered more adherent to the HMA substrate. These results confirm the initial hypothesis suggesting a potential improvement in adhesion of the composite compared to the base photocatalyst film (TiO_2_).

### 3.3. Photocatalytic Tests

The initial gas concentrations were measured in the dark for 30 min; then, the lamp was turned on, and the concentrations were measured for 30 min more. Table 4 shows the results of the photocatalytic tests.

The photocatalytic NO_2_ conversion efficiency of TiO_2_ observed in this study (averaging 17%) was slightly lower than the typical range reported in the literature, which varies between approximately 20% and 30% [36,46,55,74,75]. A likely reason for this reduction could be the extremely high gas concentration introduced into the reactor (500 ppm), as typical concentrations used in the literature for evaluating photocatalytic pavements are in the range of 1 ppm or ppb. This observation aligns with the findings of Mikyskova et al. [69], who evaluated TiO_2_ (Degussa P25) and reported a reduction in NO_2_ conversion efficiency from 70% to 12% when pollutant concentration increased from 0.1 ppm (100 ppb) to 1 ppm.

However, testing at high NO_2_ concentrations can provide insights despite the challenges posed to photocatalytic efficiency. Under these conditions, the material surfaces were susceptible to saturation, which may have limited NO_2_ adsorption at active sites, essential for photocatalysis. High concentrations may also increase the presence of intermediates like NO species, which can compete for active sites and reduce conversion rates [30]. Additionally, elevated gas levels can intensify electron–hole recombination, restricting the formation of reactive species necessary for oxidation, while the buildup of byproducts could further inhibit photocatalytic activity [76]. Despite these effects, studying high-concentration conditions highlighted the material’s behavior under more extreme pollution scenarios, offering an assessment beyond the typical low-concentration conditions in photocatalytic pavement research.

Furthermore, increasing the concentration of both TiO_2_ and CS-TiO_2_, from 2.5 to 5.0 g/m^2^, reduced the NO_2_ conversion. This behavior was also observed by other researchers who studied the effect of photocatalyst concentration on the surface of pavement and other construction materials [54]. This effect is generally attributed to the overlapping of photocatalyst particles, increased agglomeration, and a consequent reduction or even blockage in radiation absorption by the photocatalyst, leading to no additional efficiency gain.

It was also observed that adding CS to TiO_2_ did not enhance NO_2_ conversion efficiency under UV radiation. A reduction in photocatalytic activity of CS on TiO_2_ under UV light has also been reported in studies by [37,76], explained as a “UV protection” or “UV barrier” effect. This behavior is beneficial and useful, particularly in applications that combine the biological properties of CS with the inorganic properties of TiO_2,_ such as fabric modifications, packaging, and anti-acne sunscreens [37,77,78].

However, the CS-TiO_2_ composite demonstrated approximately 14% NO_2_ conversion efficiency in the visible light range. For photocatalytic field applications or experimental sections that rely on natural sunlight to photoactivate semiconductors, this additional absorption benefit, in the visible region, could make the composite relevant for reducing the presence of this pollutant in ambient air.

## 4. Conclusions

This study investigated the potential of HMA functionalized with a chitosan–TiO_2_ composite (CS–TiO_2_) to mitigate high concentrations of NO_2_ and improve the adhesion of the photocatalytic film to the asphalt mixture surface. To this end, HMA specimens were functionalized with either TiO_2_ or CS–TiO_2_, applied at two surface concentrations (2.5 and 5.0 g/m^2^). The photocatalytic efficiency of each material was tested under both visible and UV light conditions. The performance of these materials was successfully characterized using an adapted photocatalytic reactor designed to simulate real-world conditions and measure pollutant degradation efficiency. Low-cost NO_2_ sensors and passive samplers integrated with the system provided an accessible and practical solution for reaction monitoring. Finally, the adhesion of the photocatalytic film to the asphalt mixture surface was assessed, showcasing potential enhancements in the wearing performance of the tested alternatives.

The results indicated that lower photocatalyst concentrations (2.5 g/m^2^) provided the specimen with better homogeneity and coverage efficiency. It was observed that applying 2.5 g/m^2^ of photocatalysts resulted in a higher NO_2_ conversion rate under UV radiation for all photocatalysts and under visible light for CS-TiO_2_.

Another significant finding was the difference in performance between TiO_2_ and CS-TiO_2_ under different lighting conditions. CS-TiO_2_ showed lower NO_2_ conversion efficiency under UV light compared to visible light, possibly due to partial UV blocking by CS. However, from an adhesion perspective, CS-TiO_2_ formulations demonstrated better adhesion to the HMA, resulting in less material loss.

These results demonstrate that photocatalyst concentration and dopant choice are critical factors for optimizing photocatalytic efficiency and durability on pavement. CS-TiO_2_ functionalization showed potential for improving TiO_2_ adhesion and ensuring consistent performance, especially in environments with visible radiation.

To further advance the application of photocatalytic pavements, future studies could focus on:

- Long-term Environmental Durability: Testing CS-TiO_2_-functionalized pavements under prolonged exposure to UV radiation and in real-world conditions.
- Scaling and Application Techniques: Investigating methods for scaling the application of CS-TiO_2_, such as mechanized spray systems.
- Evaluation of byproducts of the photocatalytic NO_2_ degradation reaction using CS-TiO_2_.
- Enhanced Photocatalytic Formulations: Exploring additional dopants or composites that could further extend the photocatalytic activity of TiO_2_ to the visible light range.
- Economic and Environmental Impact Analysis: Conducting life-cycle analyses to evaluate the economic feasibility, material costs, and potential environmental impacts associated with the production, application, and maintenance of CS-TiO_2_-coated pavements.
- Photocatalyst Regeneration: Studying potential methods for regenerating the photocatalytic surface of the pavement, which could extend the material’s useful life and maintain its efficiency in pollutant abatement over time.

## Figures and Tables

**Table 1:** Specifications of the AQM sensors used in the experiment.

Sensor Type	Parameter	Specification
BME280	Temperature	−40 to +85 °C ± 1.0 °C
	Humidity	0 to 100% ± 3%
	Pressure	300 to 1100 hPa ± 1.0 hPa
MiCS-6814	NO_2_	0.05 to 10 ppm

**Table 2:** Experimental Design Matrix for Evaluating NO_x_ Degradation Efficiency in Photocatalytic Asphalt Mixtures.

Run	Control Factor			Random Run Order
	Photocatalyst	Wavelength	Application Rate (g/m^2^)	
1	TiO_2_	UV	2.5	2°
2	TiO_2_	UV	5.0	4°
3	CS-TiO_2_	Visible	2.5	1°
4	CS-TiO_2_	Visible	5.0	5°
5	CS-TiO_2_	UV	2.5	3°
6	CS-TiO_2_	UV	5.0	6°

**Table 3:** Adhesion Test Results of Photocatalysts on Top of Specimen Surface.

Photocatalyst	Application Rate (g/m^2^)	Pre-Tape Test	Post-Tape Test	Peeled Area (Threshold)	Peeled Area (%)
TiO_2_	2.5	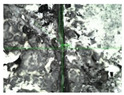	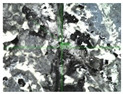	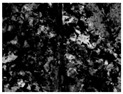	27.6 ± 2.0
	5.0	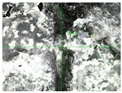	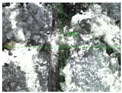	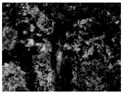	28.5 ± 1.1
CS-TiO_2_	2.5	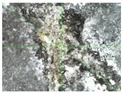	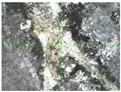	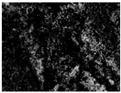	22.6 ± 1.1
	5.0	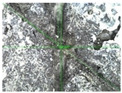	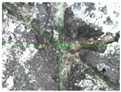	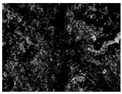	25.5 ± 0.7

**Table 4:** Photocatalytic Test Results.

Run	Control Factor			Conversion NO_2_	
	Photocatalyst	Wavelength	Rate (g/m^2^)	AQM (%)	Passive Sampler (%)
1	TiO_2_	UV	2.5	24 ± 2.1	21 ± 2.0
2	TiO_2_	UV	5.0	16 ± 1.4	7 ± 0.5
3	CS-TiO_2_	Visible	2.5	14 ± 2.8	18 ± 1.4
4	CS-TiO_2_	Visible	5.0	8 ± 2.1	16 ± 0.6
5	CS-TiO_2_	UV	2.5	15 ± 0.7	28 ± 0.7
6	CS-TiO_2_	UV	5.0	6 ± 1.5	9 ± 0.9

## Data Availability

The original contributions presented in this study are included in the article. Further inquiries can be directed to the corresponding authors.
